# The effects of intracrystalline chain diffusion on the non-linear mechanics of semicrystalline polymers

**DOI:** 10.1038/s41467-026-78014-w

**Published:** 2026-09-26

**Authors:** Rose Mary Michell, Albrecht Petzold, Thomas Thurn-Albrecht

**Affiliations:** 1 Martin-Luther-University Halle-Wittenberg, Institute of Physics, Halle, Germany; 2 Present Address: Dep. de Ciència i Enginyeria de Materials, eb-POLICOM-e-PLASCOM, Escola d’Enginyeria de Barcelona Est (EEBE), Universitat Politècnica de Catalunya, BarcelonaTech (UPC), Av. Eduard Maristany 16, Barcelona, Spain

**Keywords:** Polymers, Mechanical properties

## Abstract

Large deformability is a specific property of many semicrystalline polymers that is enabled by plastic flow going along with a restructuring of the semicrystalline morphology in combination with strain hardening caused by network forces. This ability has been linked with the intracrystalline chain diffusion (ICD), which allows chains to move within the crystals. However, the influence of ICD on the non-linear mechanics remains unclear. We here study the intrinsic non-linear mechanical properties of two model polymers, namely PEO with fast ICD and PCL without ICD by mechanical testing in plane strain compression in dependence of strain rates and temperature. For both polymers the stress could be decomposed in an elastoplastic contribution and a network contribution. Strong differences in the true stress-true strain curves became evident by comparing the rate dependence at larger deformation. The stress in PEO depends strongly on rate and temperature, indicating relaxation of the network contribution, while the stress in PCL is rate independent. Experiments for PEO with different molecular weights indicate that the relaxation affects entire chains as expected for ICD.

## Abstract (web summary)

Intracrystalline chain diffusion (ICD) allows chains to move within the crystals in semicrystalline polymers and has been linked to the deformability of such polymers but the influence of ICD on the non-linear mechanics remains unclear. Here the authors study the intrinsic non-linear mechanical properties of two model polymers with fast and without ICD.

## Introduction

Semicrystalline polymers exhibit distinctive mechanical properties, such as strength, flexibility, and toughness, that set them apart from other crystalline and amorphous materials, primarily due to their chain-like nature and their nanoscopic two-phase structure consisting of thin lamellar crystals separated by disordered amorphous layers that contain entanglements retained during crystallization^1^. More specifically, beyond the linear elastic region, upon yielding, plastic flow sets in, going along with a restructuring of the semicrystalline morphology, while at the same time, the stress-strain curves show strain hardening caused by network forces.

Starting from the 1970s, a lot of work deals with the question by which microscopic processes the semicrystalline morphology is transformed during tensile deformation from an initial isotropic spherulitic structure to a final oriented fibrillar structure. Two main processes were proposed: crystallographic slip and melting/recrystallization^2–4^. Subsequent work by the groups of Strobl^5–10^ and later Bartczak^11–14^, as well as Schrauwen^15^ extended the focus to the role of the amorphous regions and the entanglements contained therein. They used mechanical tests at constant true strain rates in tensile deformation and in compression in combination with structural investigations to analyze different stages of deformation. A recent review is given in reference^16^.

**Fig. 1:**
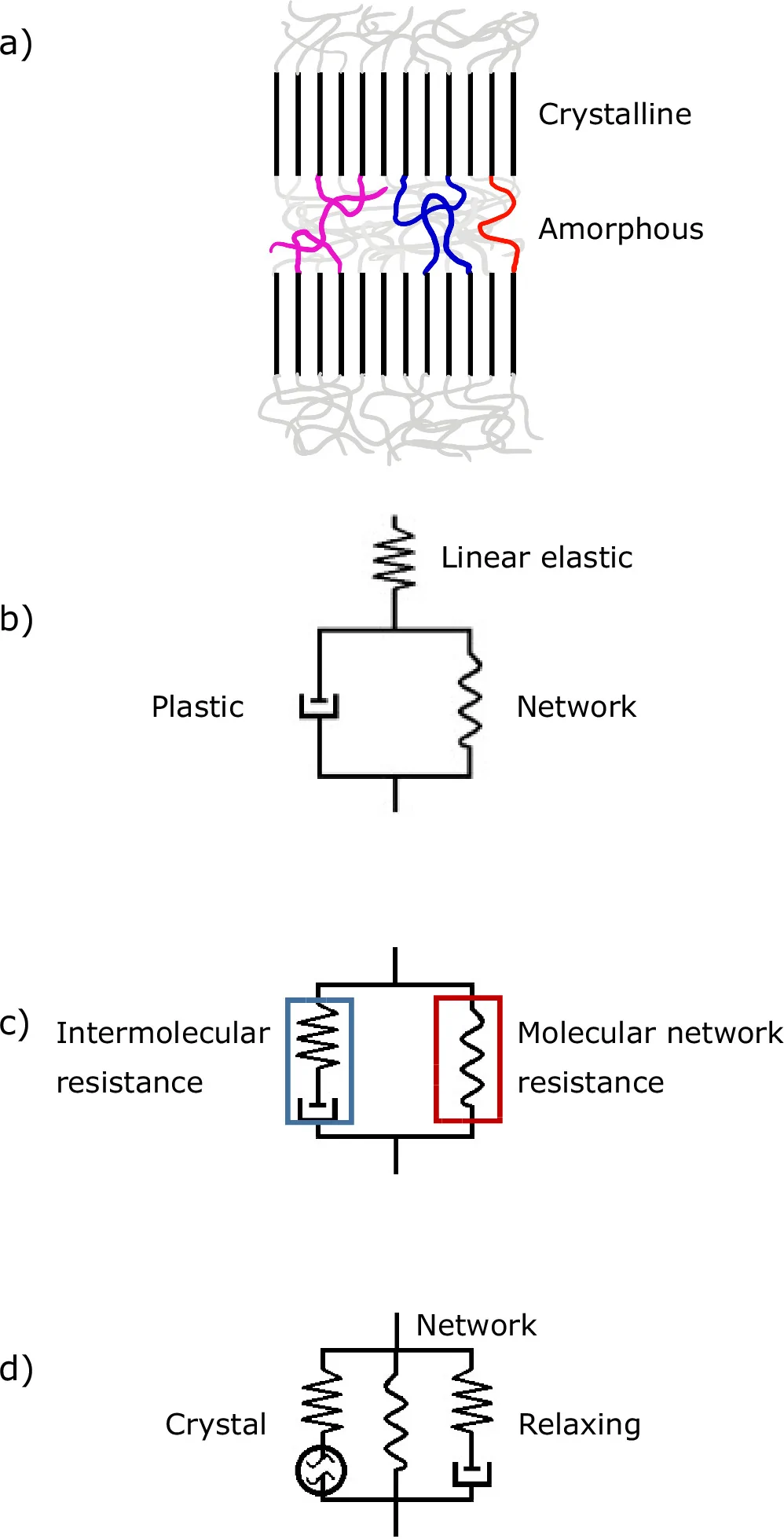
Models describing mechanical properties of semicrystalline polymers and underlying microscopic structure. **a** Sketch of the mechanically relevant nanostructural elements of semicrystalline polymers. Thin lamellar crystals with a typical thickness of the order of 10 nm (black) separated by amorphous layers are mechanically connected by tie-chains (red), entanglement loops (blue) or entanglement links (magenta). **b**–**d** Physical-based models describing the mechanical properties of semicrystalline polymers following different authors: **b** Haward and Thackray^19^, **c** Boyce et al.^21^, and **d** Strobl et al.^10^.

From all these studies, a qualitative explanation of the mechanical properties of semicrystalline polymers arises. In the linear range at small deformation, the stress is caused by a skeleton made up of small, relatively stiff crystals that are part of the spherulitic microstructure. At yield, this skeleton breaks up. The network forces showing up at larger deformation beyond the yield point are caused by the amorphous parts of the chains. However, the crystals play an important role here. Whereas in a polymer melt, the entanglements can only act as transient junction points and therefore form only a transient network, crystallization leads to the formation of a permanent network. Crystals, even if fragmented during deformation, can act as permanent junction points, on the one hand trapping the entanglements and making them permanent, on the other hand introducing new effective network strands like tie chains, entanglement loops or even more complex topological structures like entanglement links, all of them connecting different crystals (cf. Fig. 1a).

Closely related to the structural studies, numerous attempts have been made to model the stress-strain relationship of semicrystalline polymers during deformation. Two main approaches have been used, the so-called phenomenological models and physics-based models^17,18^. The former describes the data without necessarily connecting it to the microscopic polymer structure or molecular mechanism of stress generation. In contrast, the physical-based approach seeks to base the models exactly on this connection.

The latter kind of models are mostly inspired by the early model of Haward and Thackray^19^ that was initially developed for glassy polymers, but was later also applied to semicrystalline ones^20^. This model comprises three elements (cf. Fig. 1b): a linear Hookean spring, a Langevin spring, and an Eyring dashpot. The linear spring is related to the initial linear elastic part of the mechanical response. The Langevin spring is used to describe the elastic amorphous network, while the dashpot represents the plastic deformation of the crystalline skeleton. If used for the analysis of network forces at intermediate or larger strains, the strain of the Hookean elastic spring is typically neglected, leading to results that are equivalent to the model by Boyce et al. discussed next^21^. They interpreted the response of semicrystalline polymers as resulting from two parallel components; one represents the intermolecular resistance to deformation, which describes the elastoplastic contributions of the crystalline skeleton, whereas the second is due to the orientation and stretching of the molecular network, which causes rubber-like forces under large deformation. (cf. Fig. 1c). Based on this approach, many modifications have been developed; Fig. 1c shows the simplest version without relaxing elements in the network branch^18^. The stress resulting from this parallel arrangement of the elements is described by a differential equation that can be solved analytically for simple deformations^22^.

Based on a detailed analysis of the elastic and plastic contributions to deformation, in which they found deviations from ideal elastoplastic behavior, Strobl and co-workers introduced a further modification, cf. Fig. 1d^10^. This model incorporates an empirical element describing the “finite plasticity” of the crystalline skeleton, a network element and a relaxation branch. The latter is taken to describe in a combined way relaxations occurring in the crystalline as well as the amorphous parts of the sample, whereby the network itself is assumed to remain intact during deformation.

A key characteristic of semicrystalline polymers is the existence of a crystalline relaxation process, the so-called α_c_–relaxation. This process is caused by mobile conformational defects in the crystalline phase and leads to intracrystalline chain diffusion (ICD)^23,24^. Within certain limits, the time scale of the fundamental conformational dynamics as well as that of the resulting transport of chains between crystalline and amorphous phases can be measured by solid-state NMR. It typically shows an Arrhenius-type temperature dependence and varies greatly between different polymers. Recent studies showed that this process is very important for the formation of the semicrystalline morphology, as the interplay of lateral crystal growth and crystal thickening enabled by ICD determines the crystal thickness and the crystallinity to a large extent^25,26^. Furthermore, there are strong indications that part of the entanglements existing in the melt prior to crystallization are dissolved during crystallization in so-called crystal-mobile polymers, i.e., semicrystalline polymers with fast ICD^27,28^. In other materials, the process is so slow or even non-existing, that it plays no role during primary crystallization. These are the so-called crystal-fixed polymers.

In addition, this relaxation process couples also to the linear mechanical properties and shows up, e.g., in the dynamic shear modulus^23,24,29^. The storage modulus of linear PE, e.g., at frequencies below and above the α_c_–relaxation, differs by about an order of magnitude^30,31^. However, studies on the effect of ICD on the non-linear mechanical properties are scarce. Hu and Schmidt-Rohr reviewed the presence of this relaxation in different semicrystalline polymers and showed that it is a requirement for ultradrawability, i.e., large tensile deformability of semicrystalline polymers with reduced entanglement concentration^23^. Temperature-dependent differences in structural changes during deformation in polypropylene and polyethylene have been observed and linked to the α_c_-relaxation^32,33^. The latter reference also reports mechanical measurements, but without closer analysis, it focuses on the analysis of cavitation phenomena at large strain. Also, the poor creep properties of UHMWPE fibers are a consequence of the intracrystalline mobility and ICD^34,35^. Finally, chain diffusion in the crystal, the required forces and energy dissipation have also been measured on a microscopic scale by single-molecule force spectroscopy in the AFM^36^.

Despite these observations, quantitative investigations and a comprehensive analysis connecting deformation, ICD, and the different stress contributions are missing. Furthermore, the majority of experiments are based on the most common semicrystalline polymers, polyethylene and polypropylene, which are both crystal-mobile. To identify the effects of ICD has therefore been difficult.

In this study, we therefore investigate the intrinsic mechanical properties of two model polymers: poly(ε-caprolactone) (PCL), which serves as an example of a polymer with slow ICD, and poly(ethylene oxide) (PEO), which exemplifies a polymer with fast ICD^25^. Using plane strain compression geometry, we measured true strain and true stress under deformation with a constant true strain rate. We focus on the region of larger deformation beyond the yield point, where the amorphous network dominates the mechanical response, as the effects are largest in this region, as shown below. In this way, we could identify fundamental differences in the properties of PEO and PCL that are related to the ICD, offering new insights into the understanding of the mechanical performance of semicrystalline polymers in general.

## Results

To measure the intrinsic mechanical properties of the polymers under study, we performed nonlinear mechanical tests in plane strain compression geometry. Supplementary Fig. 1 shows a schematic of the custom-made sample holder employed for the experiments in a testing machine. During compression, one direction is constrained, and deformation occurs only in two directions; this geometry leads to a pure shear deformation^37^. One significant advantage of this setup is that the force is applied over a constant area, which allows a direct determination of the true stress. Moreover, the compression geometry minimizes the formation of cavities and suppresses necking. In this way, it is therefore feasible to study the behavior of semicrystalline polymers with reduced influence from the instabilities common in tension^38,39^.

**Fig. 2:**
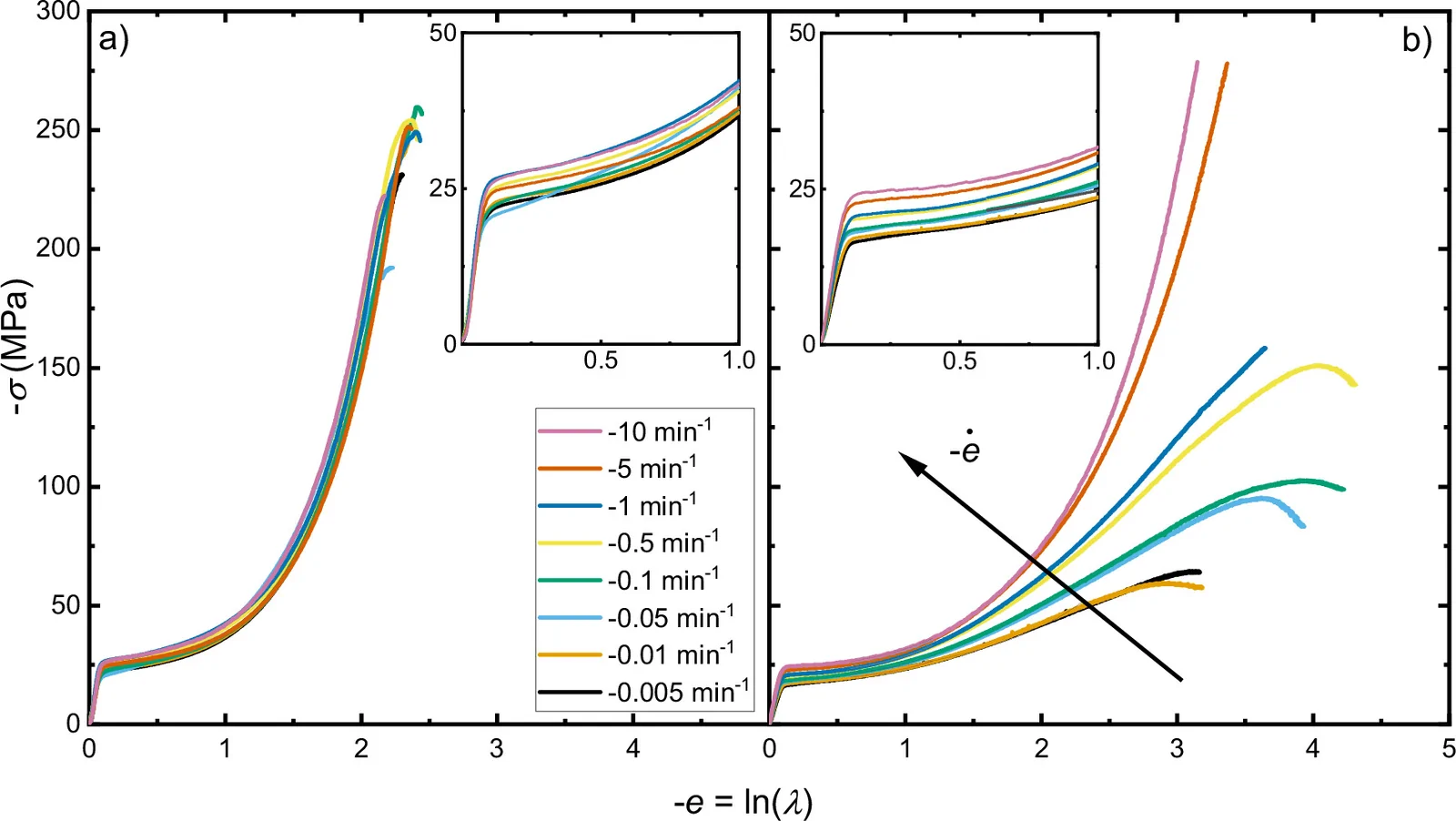
Exemplary continuous true stress (*σ*) vs. true strain (*e*) curves. **a** PCL and **b** PEO100 measured at a temperature of 30 °C. Measurements were taken at constant true strain rates in plane strain compression as indicated. Arrows indicate how the data shift with increasing true strain rate. The decreasing stress at the final part of the curves at large true strain indicates failure of the sample. For a detailed description of the samples and variables used, refer to the method section. The insets show the initial part of the curves, focusing on the range around the yield point.

A first series of measurements with different true strain rates was performed at a temperature of 30 °C on two samples of moderate molecular weight, PCL and PEO100, with molecular weights of 80 kg/mol and 100 kg/mol, respectively. The resulting stress-strain curves are shown in Fig. 2.

The general shape of the curves is similar for both polymers and corresponds to the known behavior of a semicrystalline polymer above *T*_*g*_. There is an initial linear part, described by Hooke’s law, followed by a change in slope around the yield point that indicates the onset of plastic flow and permanent deformation. The yield stress shows only a slight increase with increasing true strain rate ($\dot{e}$). This effect is a bit stronger for PEO100 than for PCL (cf. inset in Fig. 2). However, at larger values of strain, the two polymers behave very differently. While the stress curves for PCL remain more or less unchanged with the rates of deformation, PEO100 exhibits much weaker strain hardening with decreasing deformation rate. Furthermore, reducing the rate results in a decrease in the stress at break for PEO100. For the highest true strain rates ($\dot{e}$ = −10 and −5 min^−1^), PEO100 did not break during the test; the required stress exceeded the stress limits of the setup. Since the differences between the two polymers occur primarily beyond the yield point, we will focus in the following on the analysis of this part of the stress-strain curves.

### Analysis of stress-strain curves

For the analysis of stress-strain curves, we use the simple model depicted in Fig. 1c as a basis, since it avoids the restrictive and at high strain unphysical assumptions of the Haward-Thackray model mentioned above. Instead, it leads to a straightforward expression for the total stress and allows a simple semiquantitative analysis of experimental data. According to the parallel arrangement of the two elements, the individual stresses add up, resulting in  the following expression for the total stress.

1 $$\sigma={\sigma }_{{ep}}+\,{\sigma }_{n}$$

Here *σ*_*ep*_ is an elastoplastic contribution that describes the elastic response of the material at small strain and goes over into a plastic contribution at larger strains, whereas *σ*_*n*_ is the network stress that describes the strain hardening at larger strains. In the elastoplastic branch, the strain of the elastic element and the plastic element add up. This contribution is related to the crystalline fraction of the material and the corresponding experimental parameters, namely the modulus and the yield stress that goes over in a plastic stress at larger strain, typically increase with crystallinity^5,9^. At a constant deformation rate, beyond the yield point the Hookean spring remains at a constant elongation and the increasing deformation is realized by the Eyring dashpot resulting in the following expression for the stress^10,40^.

2 $${{\sigma }_{{ep}}=\sigma }_{o}{\sinh }^{-1}\frac{\dot{e}}{{\dot{e}}_{o}}$$

Here reference stress *σ*_*o*_ and reference strain rate ${\dot{e}}_{o}$ are the parameters describing the Eyring dashpot. Equation 2 simplifies to

3 $${{\sigma}_{ep} \approx {\sigma} }_{o}{{\mathrm{ln}}}\frac{\dot{e}}{{\dot{e}}_{o}}\,$$

for $\dot{e}\gg {\dot{e}}_{o}$, which is the appropriate limit for plastic flow^40^. In consequence, *σ*_*ep*_ is constant beyond yield for a given true strain rate $\dot{e}$ and can be subtracted from the total stress. *σ*_*n*_, on the other hand, can be modeled by an appropriate constitutive equation for rubber elasticity. In the simplest case, the neo-Hookean model is used, where an affine deformation is assumed, and the chains are described as Gaussian chains. In this case for plane-strain compression

4 $${\sigma }_{n}={G}_{n}\left({\lambda }^{2}-1/{\lambda }^{2}\right)$$

with the extension ratio *λ* (cf. Eq. (8)) and the network modulus

5 $${G}_{n}={\nu }_{e}{k}_{b}T .$$

Here ν_e_ is the number of network strands per volume or more general stress transmitters in the case of semicrystalline polymers^27^. The neo-Hookean model does not account for additional strain hardening caused by the finite extensibility of the chains. Bartczak suggested using the 8-chain model, a well-established rubber model, to model these effects in semicrystalline polymers^41,42^. The model of Fig. 1c alone, of course, does not yet include any relaxation of network stresses. For that purpose, one or more additional relaxing elements would need to be introduced^21,43^.

### Separation of stress components

**Fig. 3:**
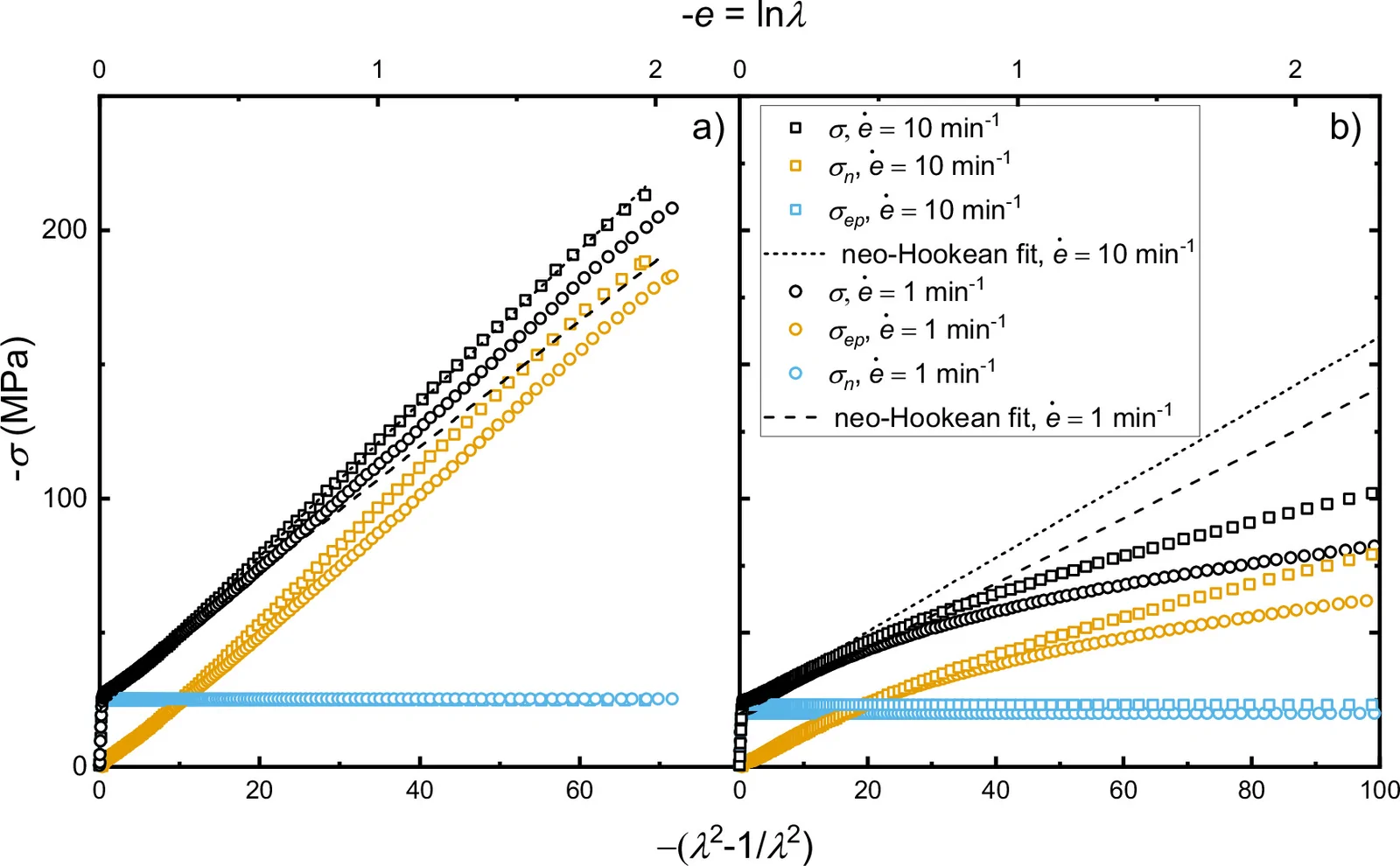
Exemplary Haward-Thackray plots. **a** PCL and **b** PEO100. Data measured at 30 °C and $\dot{e}$ values of 1 (black circles) and 10 min^−1^ (black squares) are shown together with a fit based on the neo-Hookean expression (broken and dashed lines), leading to a separation into the elastoplastic stress *σ*_*ep*_ (blue) and the remaining network stress *σ*_*n*_ (brown). The number of data points was reduced for better visibility.

To separate the elastoplastic and network contributions to the stress, we plot the experimental data in the so-called Haward-Thackray plot, σ vs *λ*^2^−1/*λ*^2^. According to Eqs. (1, 3 and 4), the elastoplastic contribution is constant beyond yield, while the network stress shows up as a straight line with positive slope if neo-Hookean behavior applies. In this way, it is possible to separate the two contributions with a linear fit of the data in the neo-Hookean range, i.e., small deformations of the network^5,44^. This procedure is illustrated for exemplary data sets of both polymers in Fig. 3, showing the individual stress contributions in different colors.

**Fig. 4:**
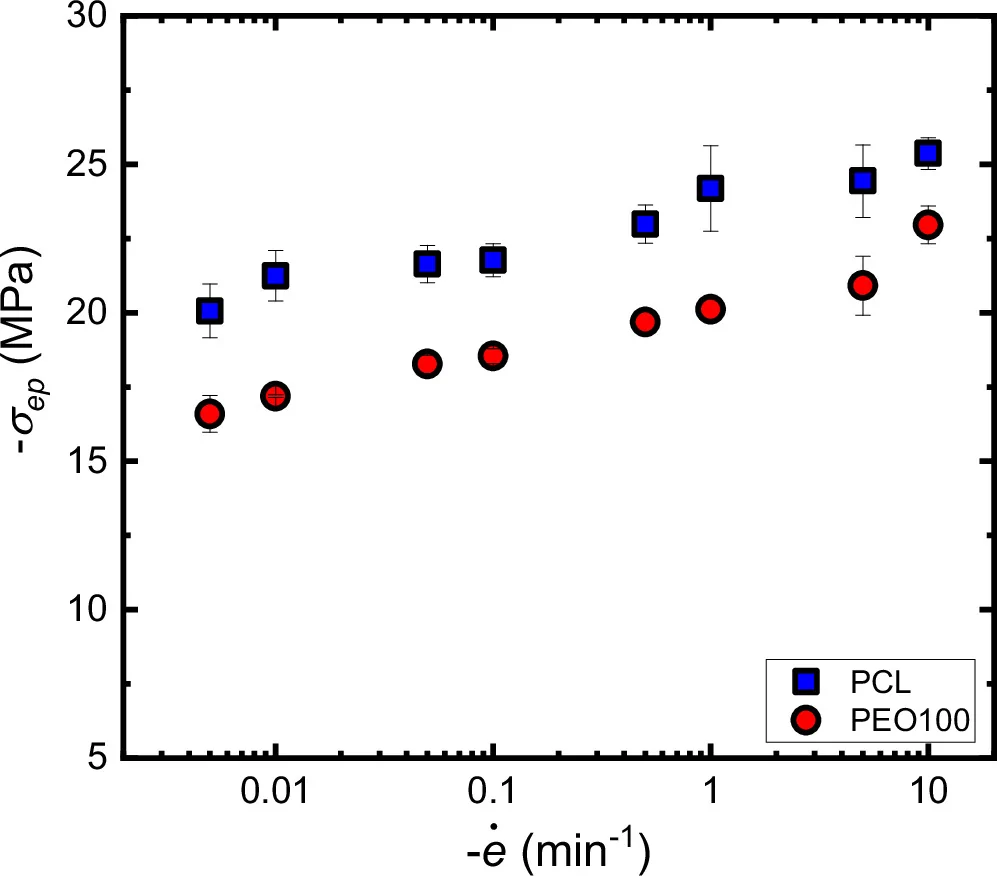
Elastoplastic stress *σ*_*ep*_ versus true strain rate for PEO100 and PCL as resulting from the separation procedure illustrated in Fig. 3. The logarithmic dependence confirms the consistency of the analytical approach. The values shown are averages of the fit parameters resulting from three independent measurements with the corresponding statistical error estimate.

To check the consistency of this approach, we plot the resulting values of *σ*_*ep*_ vs. the strain rate in Fig. 4. The expected logarithmic dependence, cf. Eq. (3), is obvious^10,27^.

Interestingly, *σ*_*ep*_ is consistently higher for PCL than for PEO100, even though PEO100 has a higher crystallinity (42% for PCL and 80% for PEO100 based on the DSC first heating scan of the as-molded samples). The question if this difference is related to the presence of fast ICD in PEO100, facilitating the plastic deformation of PEO, or an independent property of PEO crystals, cannot be answered based on the data shown here. An in-depth analysis of this effect is therefore beyond the scope of this work.

**Fig. 5:**
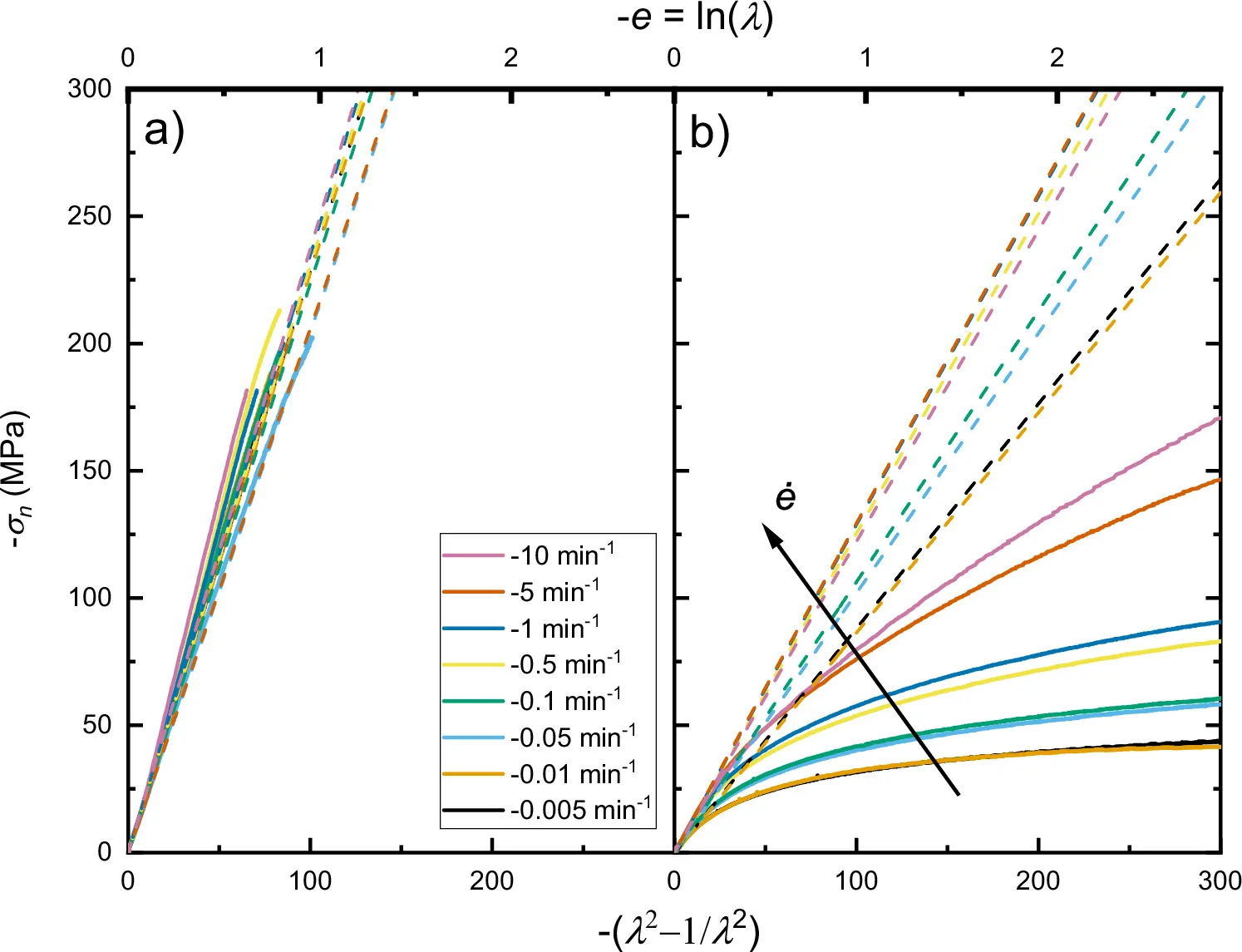
Exemplary curves of the network stress *σ*_*n*_ in a Haward-Thackray plot. **a** PCL and **b** PEO100. The extrapolated fits based on the neo-Hookean model are shown as dashed lines.

We can now focus on the network contribution *σ*_*n*_, shown in Fig. 5. It is obviously this contribution that is responsible for the differences in the stress-strain curves of crystal-fixed PCL and crystal-mobile PEO. While for PCL the measured stress curves lie slightly above the extrapolated neo-Hookean curves, there seems to be a relaxation present in PEO that causes a substantial, rate-dependent negative deviation below the neo-Hookean force law.

### Network stress in PCL as a crystal-fixed polymer

Let us first focus on PCL. The measured values follow largely the extrapolated neo-Hookean prediction with some positive deviation at larger deformation values. Such a behavior corresponds qualitatively to the non-linear mechanical properties of a rubber-like material. In that case, the deviations at larger stresses would be caused by the limited extensibility of the stretched network strands.

**Fig. 6:**
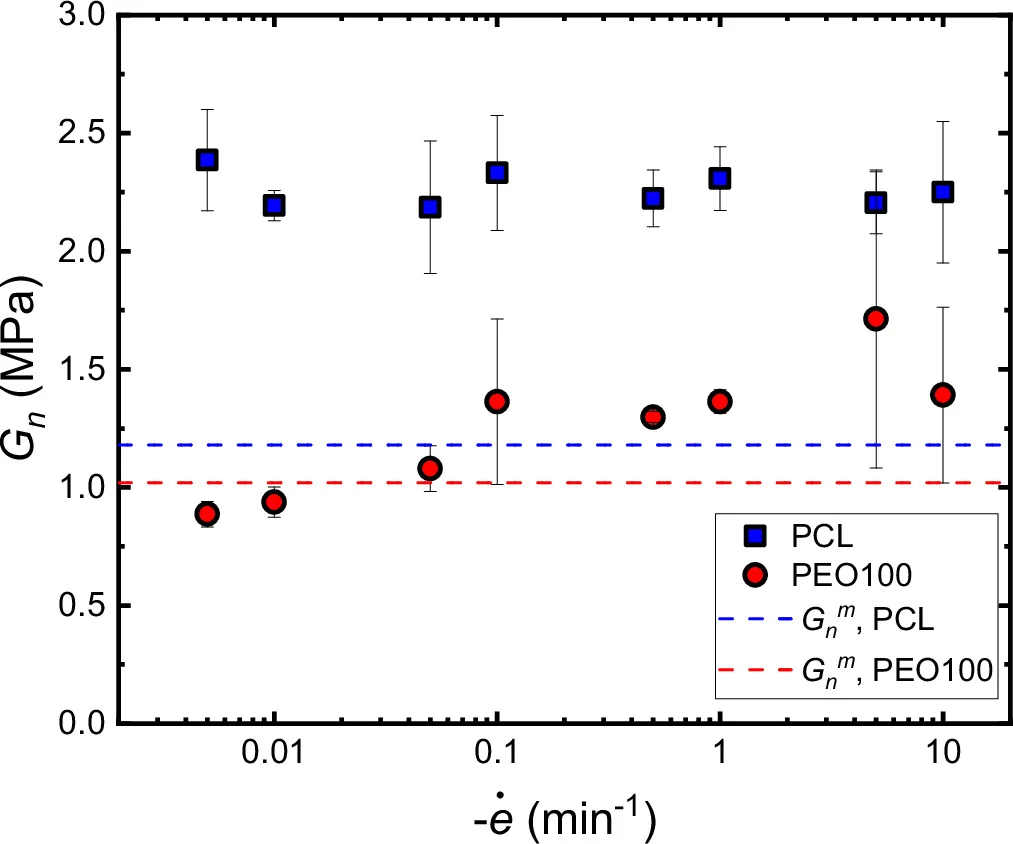
Initial network modulus versus true strain rate for PCL and PEO100 at 30 °C. Dashed horizontal lines show for comparison the plateau modulus ${G}_{n}^{m}$ of PCL^28^ and PEO^52^ in the melt state. The values shown are averages of the fit parameters resulting from three independent measurements with the corresponding statistical error estimate.

Taking into account the deviations at large stresses, we call *G*_*n*_ the initial network modulus; in the literature, it is also sometimes called strain-hardening modulus. The measured values are shown in Fig. 6; different from the elastoplastic contribution, they show no rate dependence for PCL, indicating an elastic character of the involved rubber forces. It is informative to compare the absolute values of *G*_*n*_ with the plateau modulus of a PCL melt, shown in Fig. 6 as a horizontal blue dashed line.

If the junction points of the network responsible for the rubber elastic forces consisted only of the entanglements in the amorphous regions, the two values should coincide^15,27^. However, our results show that the value in the semicrystalline state of PCL is larger by nearly a factor of two, suggesting that small crystallites act as additional junction points with tie chains in between. In this context, it is also of relevance that we previously showed that in PCL as a crystal-fixed polymer, entanglements are not dissolved but rather redistributed to the amorphous phase during crystallization^45^.

As mentioned above, the full stress-strain curve of semicrystalline polymers measured in plane strain compression, including additional strain hardening beyond the neo-Hookean model, has been modeled by Bartczak using the 8-chain model^41^. This model describes finite extensibility effects with an inverse Langevin function and contains as an additional parameter the number of Kuhn segments N between junction points. Using the Padé approximation to the inverse Langevin function, the 8-chain model results in the following expression for the network stress as a function of true strain^42,46^.

6 $${\sigma }_{n}(e)=\frac{{G}_{n}}{3}\cdot (\exp (2e)-\exp (-2e))\cdot (\frac{9N-\exp \left(2e\right)-\exp \left(-2e\right)-1}{3N-\exp \left(2e\right)-\exp \left(-2e\right)-1})$$

**Fig. 7:**
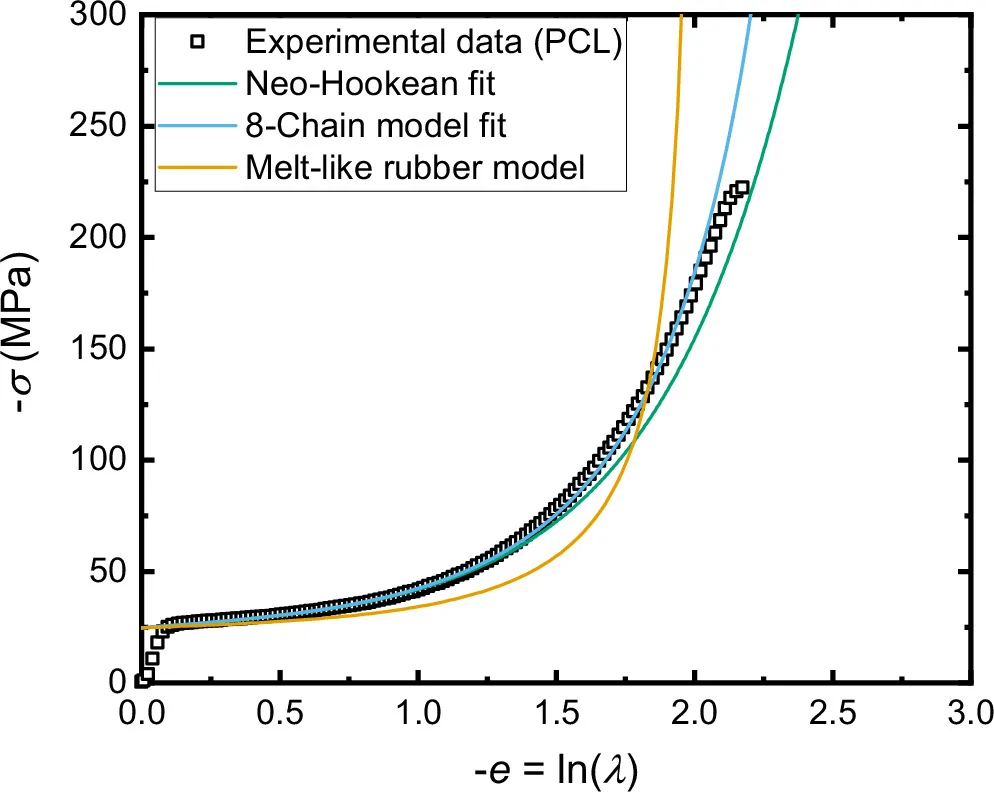
Exemplary curve of network true stress versus true strain for PCL at 30 °C and 10 min^−^^1^ together with different model curves. Fit curves based on the 8-chain model and Neo-Hookean models are displayed. A further model curve based on the 8-chain model for a rubber, with parameters typical for a PCL melt (*G*_*n*_ = 1.18 MPa and *N* = 20), is presented for comparison^28^. The number of data points was reduced for better visibility.

As visible in Fig. 7, in this way, the measured curve is well described up to the region where the sample starts to break. Here, the constant elastoplastic stress *σ*_*ep*_ is added. However, it is important to recognize that the resulting number of Kuhn segments between junction points *N* is unrealistically large (*N* ≈ 100), about a factor of five higher than the number of Kuhn segments calculated from the molecular weight between entanglements in the melt (*N* ≈ 20)^28^. This result is the more remarkable given the fact that *G*_*n*_ is about a factor of two higher than the plateau modulus of the melt ${G}_{n}^{m}$. Based on this value, one would expect a smaller, not a greater value N.

Qualitatively, this discussion reveals that the observed strain hardening is very weak. A rubber whose initial network modulus *G*_*n*_ and length of network strands *N* has the same values as the plateau modulus ${G}_{n}^{m}$ and the length of the entanglement strands *N*_*e*_ as a PCL melt would exhibit a much stronger strain hardening, as illustrated in Fig. 7. This comparison indicates that the network that causes the stress in semicrystalline polymers is not fixed, it evolves during deformation. It is well known that the semicrystalline morphology transforms during deformation from an originally isotropic spherulitic structure to an oriented fibrillar structure. Crystallites act as junction points of an effective network, and of course, the deformation-induced breakup and reorganization of the semicrystalline morphology happens in such a way that the strands of this network release stress. As a result, the strain hardening becomes weaker than expected from an extrapolation of the former parts of the stress-strain curve. The fit parameters *G*_*n*_ and *N* should therefore only be considered as effective parameters that describe the stress-strain curve, rather than a real network that would remain unchanged during deformation. Nevertheless, the interpretation of *G*_*n*_ as the modulus of the initial network still seems reasonable. Note also that the discussed changes of the network structure are obviously rate-independent, at least for the range covered in our experiments.

### Network stress in PEO as a crystal-mobile polymer

Taking the results obtained for PCL and their discussion above as a reference, we can now proceed with the analysis, including the results obtained for PEO. During the discussion of Fig. 5, we suggested that the strong rate-dependence of the stress-strain curves is caused by a relaxation process that is not present in PCL.

Let us first look at the initial network modulus *G*_*n*_ of PEO shown in Fig. 6. In contrast to PCL, the values for PEO100 display a logarithmic dependence on $\dot{e}$. Furthermore, the values are lower and indeed similar to the plateau modulus of a PEO melt, ${G}_{n}^{m}$. We interpret both facts as a consequence of the ${\alpha }_{c}$-relaxation. The rate dependence is obviously indicative of a friction-generating element. The reason for the lower values of *G*_*n*_ can be twofold. On the one hand, the high mobility of the chains in the crystals reduces their effectiveness as junction points of the network, and this is the more the case the slower the deformation happens. On the other hand, a certain amount of entanglements could be resolved during the crystallization in crystal-mobile PEO, depending on molecular weight and crystallization rate.

To further analyze the effects of the ${\alpha }_{c}$-relaxation we use the concept of the Mooney ratio (${G}_{n}^{*}$), which for plane strain compression reads as

7 $${G}_{n}^{*}(\lambda )=\frac{{\sigma }_{n}}{{\lambda }^{2}-1/{\lambda }^{2}}=\frac{\sigma -{\sigma }_{{ep}}}{{\lambda }^{2}-1/{\lambda }^{2}}.$$

**Fig. 8:**
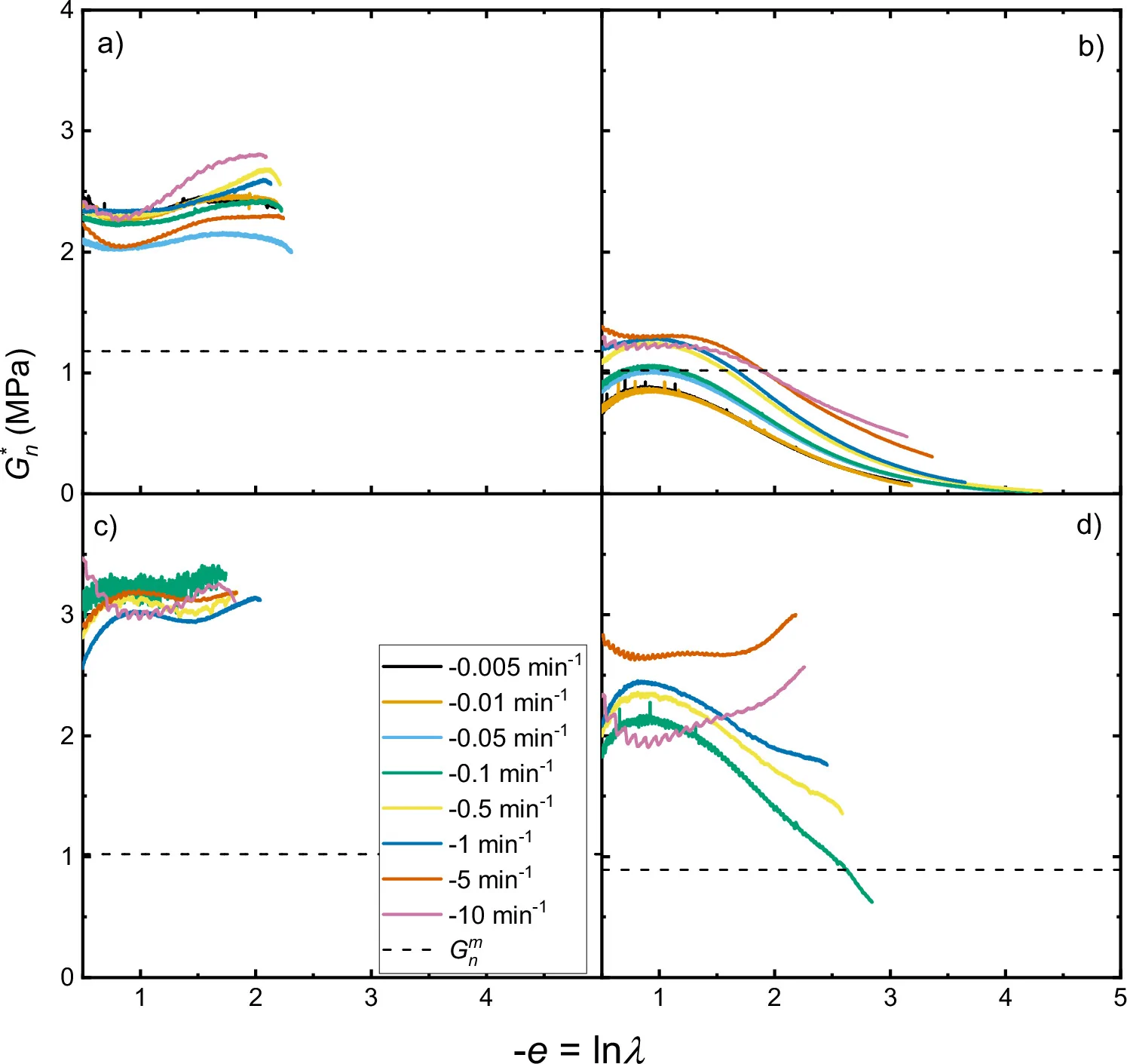
Mooney ratio vs. true strain. **a** PCL and **b** PEO100 at 30 °C, **c** PCL and **d** PEO100 at −10 °C for different true strain rates. The original stress-strain curves are shown in Supplementary Fig. 2.

The Mooney ratio is commonly used to analyze and describe the complex stress-strain dependence of soft rubbers^47–49^. For a stress-strain curve that follows the neo-Hookean expression (Eq. (6)), ${G}_{n}^{*}$ is a constant and equal to the initial network modulus *G*_*n*_. For additional strain hardening, e.g., due to finite extensibility of chains, ${G}_{n}^{*}$ would increase along the stress-strain curve; a relaxation of the network shows up as a decrease. Fig. 8a, b) show the same data as Fig. 5a, b) and illustrate that in this presentation, both effects discussed above come out clearly. The initial network modulus that corresponds to the initial plateau of ${G}_{n}^{*}$ is larger for PCL, while the further course of the curves has opposite trends for PCL and PEO. The increase for PCL reveals strain hardening beyond the neo-Hookean model, and the decrease for PEO is indicative of stress relaxation during deformation. Note that the first part of the stress-strain curves, corresponding to the linear Hookean behavior up to yield, cannot be analyzed in this presentation; only the data with true strain beyond −0.5 are shown. To further test our hypothesis that the differences in the mechanical properties, as they show up in the stress-strain curves, are caused by the ICD, we performed temperature-dependent measurements. As the ${\alpha }_{c}$-relaxation is an activated process, we would expect decreasing relaxation effects at lower temperatures for PEO.

Figure 8c, d) show the values of ${G}_{n}^{*}$ at a temperature of −10 °C for PCL and PEO100. The shape of ${G}_{n}^{*}$ for PCL at this lower temperature is very similar to the one at 30 °C, namely a constant followed by a weak increase at larger deformation beyond $\left|e\right|\gtrsim 1.2$. These effects were discussed above. However, additionally, there is an increased value of *G*_*n*_ at the lower temperature. Given that PCL, as a crystal-fixed polymer, exhibits insertion crystallization upon cooling, the appearance of additional crystalline junctions points in the network is expected^25^.

However, most interesting are the combined temperature and rate-induced changes for PEO. At a temperature of −10 °C and small |$\dot{{e|}}$, the behavior is similar to the one found at 30 °C. However, for higher $\dot{e}$, (−10 and −5 min^−1^), the curves resemble the ones of PCL. *G*_*n*_ shows a higher value, and there is no stress relaxation at higher *e*. That implies that at a temperature of −10 °C, the range of external deformation rates crosses the time scale of the ICD, so that the stress can relax during slow deformation but not during fast deformation. For the combination of high rates and low temperature, the effect of the ICD is suppressed, and the crystals now act as effective junction points of the amorphous network, also in PEO.

**Fig. 9:**
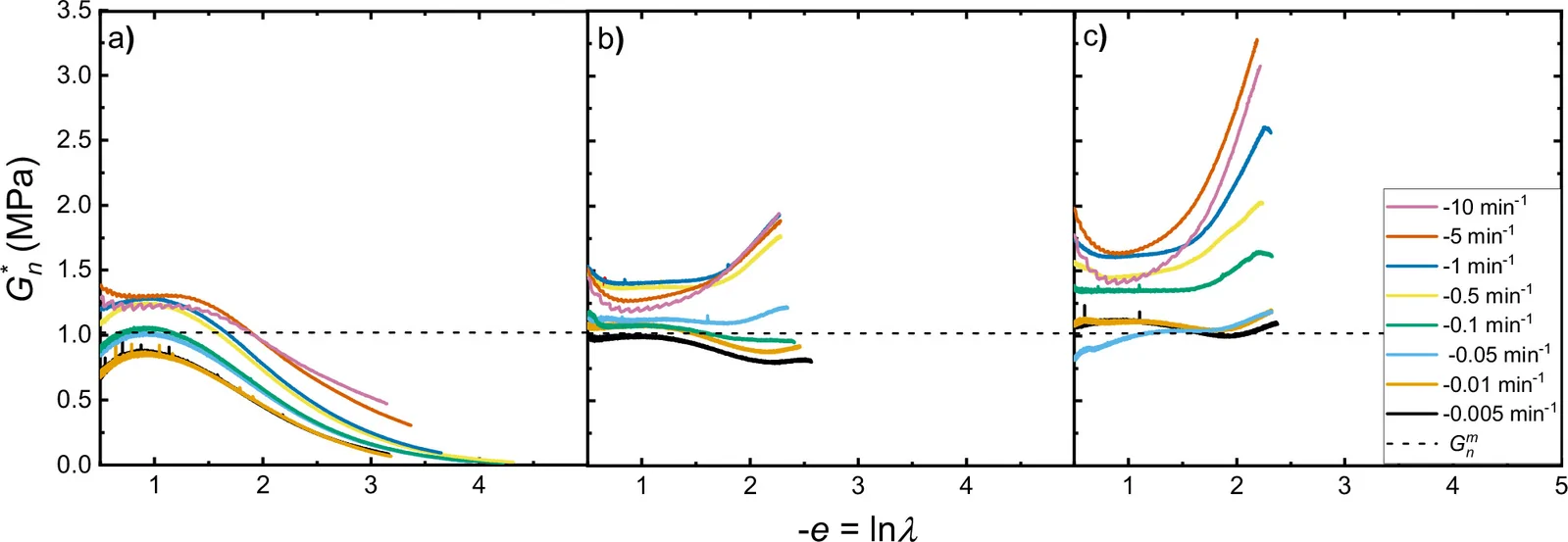
Mooney ratio vs. true strain for PEO of different molecular weight. **a** PEO100 **b** PEO400, and **c** PEO900 measured at the indicated rates at 30 °C.

Having established the combined rate-temperature dependence of network relaxation during nonlinear deformation in PEO, it remains to be shown that the relaxation is indeed connected to the ICD, i.e., to a relaxation of the whole chain and not only to some local structural rearrangement. We can use here a similar approach as is taken to analyze the molecular dynamics in polymer melts, namely, to check the molecular weight dependence. A relaxation process involving whole chains should depend on molecular weight; a local process generally does not. We therefore performed mechanical measurements of PEO with three different molecular weights, of about 100 kg/mol, 400 kg/mol and 900 kg/mol. The corresponding ${G}_{n}^{*}$ is shown in Fig. 9, the corresponding original stress-strain data in Supplementary Fig. 3. A comparison of the data for the three molecular weights shows very clearly that with increasing molecular weight and increasing rate, there is a change in properties from relaxation to strain-hardening and increasing ${G}_{n}$, i.e. the relaxation is suppressed. Basically, with increasing molecular weight, the stress-strain curves change in a similar way as with decreasing temperature, i.e., the network relaxation obviously becomes slower, exactly what is expected for a process that involves a motion of the whole chain. Given the change in shape of ${G}_{n}^{*}$, this conclusion remains valid even if one takes into account possible static, structural effects on ${G}_{n}$. On the one hand, the entanglement concentration in the amorphous regions might increase with molecular weight, as discussed above; on the other hand, the crystallinity decreases with molecular weight.

## Discussion

In summary, the stress-strain curves of the semicrystalline polymers under study could be consistently decomposed into two contributions: the elastoplastic contribution that dominates the first part of the curve until yielding, after which this contribution remains constant, and a second one, caused by network stresses. Using this approach, we found fundamental differences in the intrinsic mechanical properties of semicrystalline polymers between a so-called crystal-fixed and a crystal-mobile polymer, that depend on the existence and/or time-scale of the α_c_-relaxation and the related ICD, and that strongly affect the network stresses. We expect these differences to be of general validity.

The network stress of PCL, a semicrystalline polymer with very slow ICD, can be described using the neo-Hookean model plus some rather weak additional strain-hardening at larger deformation. It does not show evidence of relaxation in the investigated parameter range. In contrast, the network stress for PEO is temperature- and rate-dependent, due to a relaxation that involves the entire chain across the entanglements and crystals. While these effects are strongest at large deformation, the presence or absence of an α_c_-relaxation also affects the initial network modulus or strain-hardening modulus. Entanglements as well as small crystal fragments can act as junction points of the network, whereby an active α_c_-relaxation reduces the role of the crystals. As a consequence, the crystal-fixed polymer under investigation, namely PCL, displays a significantly larger initial network modulus *G*_*n*_ than PEO, for which its value depends on temperature, molecular weight and deformation rate. Obviously, all these properties are highly relevant at large global or local deformation and on the other hand, for processing in the solid state, for example, in cold drawing. Qualitatively, ICD enables large deformations at considerably lower stresses at small deformation rates. Understanding the criteria for terminal failure, on the other hand, requires further work and is out of scope for this publication.

Future research should further check the extent to which the observed effects of ICD are indeed of general validity beyond the model polymers used. In this context, it is important to realize that polyethylene, one of the most heavily used commercial polymers, also belongs to the class of crystal mobile polymers. Furthermore, the nature of the observed relaxation after nonlinear deformation should be further clarified. A possible next step would consist of stress relaxation experiments to determine the shape, amplitude and time/temperature dependence of the relaxation. Such experiments would hopefully also provide further evidence that can be used to refine the mechanical models used for semicrystalline polymers as they are depicted in Fig. 1. It is obvious that to describe the properties of PEO, the simple version of the Boyce model shown in Fig. 1c lacks an element leading to relaxation in the network branch. On the other hand, introducing a relaxing branch in parallel, as suggested by Strobl, does also not allow a relaxation of the network as detected in our work. Furthermore, in this work, we focused on the impact of the α_c_-relaxation﻿ and ICD on the network properties, while we neglected their effects on the elastoplastic stress contribution. As mentioned above, these turned out to be weaker, but they exist as suggested before^15^.

We hope that in the long-term experiments and analyses as shown here, will provide a more fundamental understanding of material properties that are usually investigated only under special conditions of standard material testing and add in this way criteria for the suitability of different semicrystalline polymers for different applications.

## Methods

### Samples

All samples (PCL and PEO) were purchased from Scientific Polymer Products (Sp^2^, Ontario, New York, USA). The PEO samples are labeled PEO according to their molecular weight in kg/mol, given by the supplier. The molecular weight of the PCL employed was 80 Kg/mol according to the supplier.

The compression test samples were molded into the shape of a rectangular bar of 15 x 6 x 5 mm using a vacuum compression molding tool by MeltPrep (Graz, Austria). The PCL and PEO100 samples were heated in the mold to 80 °C and kept there for 20 min. Due to their higher viscosity, PEO400 and PEO900 were heated to 155 °C and also kept for 20 min. Afterward, the mold was transferred to a cooling plate with a fixed temperature of 21 °C and kept there for 5 min. The crystallinity of the samples was determined by a subsequent DSC measurement as ${{X}_{c}=\frac{{\Delta H}_{m}}{{\Delta H}_{m}^{100}}}_{c}$ using a value of ${\Delta H}_{m}^{100}={\mathrm{196,6}}$ J/g (PEO) and ${\Delta H}_{m}^{100}=157.0$ J/g (PCL) for the melting enthalpy of a sample with 100% crystallinity^50,51^. The results are given in Table 1.

**Table 1:** Sample materials with weight-averaged molecular weight, melting temperature, melting enthalpy and DSC based ﻿crystallinity

Sample	Mw/kg mol^-1^	T_m_ (°C)	ΔH_m_ (J/g)	X_c_ (%)
PCL	80	59.4	80.5	53.8
PEO100	100	67.5	153.8	78.2
PEO400	400	70.0	131.6	67.0
PEO900	900	70.1	130.8	66.5

### Mechanical testing

Mechanical measurements were performed using a universal testing machine TIRAtest 2850 (TIRA GmbH, Schalkau, Germany) equipped with a 50kN load cell and a temperature-controlled chamber TH2700 (Grip Engineering Thuemler GmbH, Nuremberg, Germany) with liquid nitrogen cooling. A homemade tool made of hardened steel with a force limit of 25kN was used for plane strain compression. The distance of the jaws was measured by two clip-on extensometers MFA 25 (Mess- & Feinwerktechnik GmbH, Velbert, Germany). The displacement of the sample was measured by the averaged value of both extensometers to avoid contributions due to the tilt of the tool and was used to control the true strain rate in situ. To reduce friction, the surface of the specimen was covered by one layer of PTFE tape with a thickness of 76 microns.

The true strain is obtained according to

8 $$e={{\mathrm{ln}}}\,\lambda={{\mathrm{ln}}}\frac{h}{{h}_{o}}={{\mathrm{ln}}}\frac{\Delta {{h}}+{h}_{o}}{{h}_{o}}$$

Here λ is the compression ratio, h_0_ is the original height of the sample, and Δh is the displacement during the measurement. The true stress is obtained as

9 $$\sigma=\frac{F}{{A}_{p}}$$

where *F* is the measured force, and *A*_*p*_ is the contact area between the plunger and the sample. *A*_*p*_ is 15 x 6 mm and constant during the entire compression test. All measurements were performed on triplicate samples. Full stress-strain curves in Figs. 2, 3, 5, 7, 8 and 9 show typical measurements. Parameters resulting from a fit to the data as in Figs. 4 and 6 are averages with the corresponding statistical error estimate.

## Supplementary information

Supplementary Information

Transparent Peer Review file

## Data Availability

The data shown in this study are available in the SADAR database (Sachsen-Anhalt Data Archive and Repository) under accession code https://doi.org/10.25673/1914118-19. All data are available from the corresponding author upon request.
