# Supplementary material for: The effects of intracrystalline chain diffusion on the non-linear mechanics of semicrystalline polymers
Source: Nat Commun. 2026 Sep 26;17:10235. doi: 10.1038/s41467-026-78014-w (PMC13615967; doi:10.1038/s41467-026-78014-w)
Supplement: Supplementary file 1 [file 41467_2026_78014_MOESM1_ESM.pdf]

# Supplementary Information

## The effects of intracrystalline chain diffusion on the non-linear mechanics of semicrystalline polymers

Rose Mary Michell[1,2]*, Albrecht Petzold[1], Thomas Thurn-Albrecht[1]*

[1] Martin-Luther-University Halle-Wittenberg, Institute of Physics, D-06099 Halle, Germany.

[2] Current affiliation: Dep. de Ciència i Enginyeria de Materials, eb-POLICOM-e-PLASCOM, Escola d'Enginyeria de Barcelona Est (EEBE), Universitat Politècnica de Catalunya, BarcelonaTech (UPC), Av. Eduard Maristany 16, 08019 Barcelona, Spain

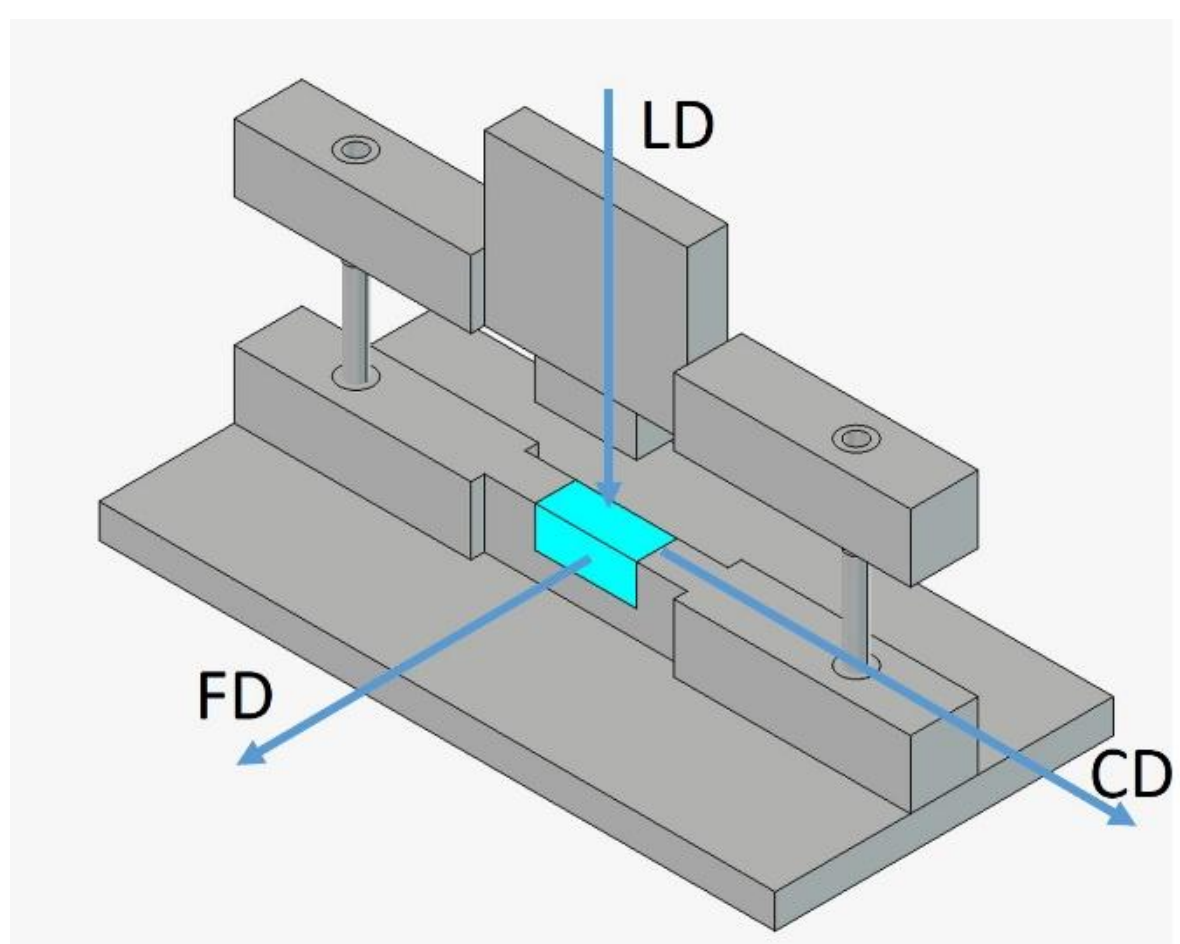


Supplementary Figure 1: Schematic representation of the tool used for plane strain compression. The arrows indicate the different directions, FD: flow direction, CD: control direction, and LD: loading direction. The blue prism represents the sample.

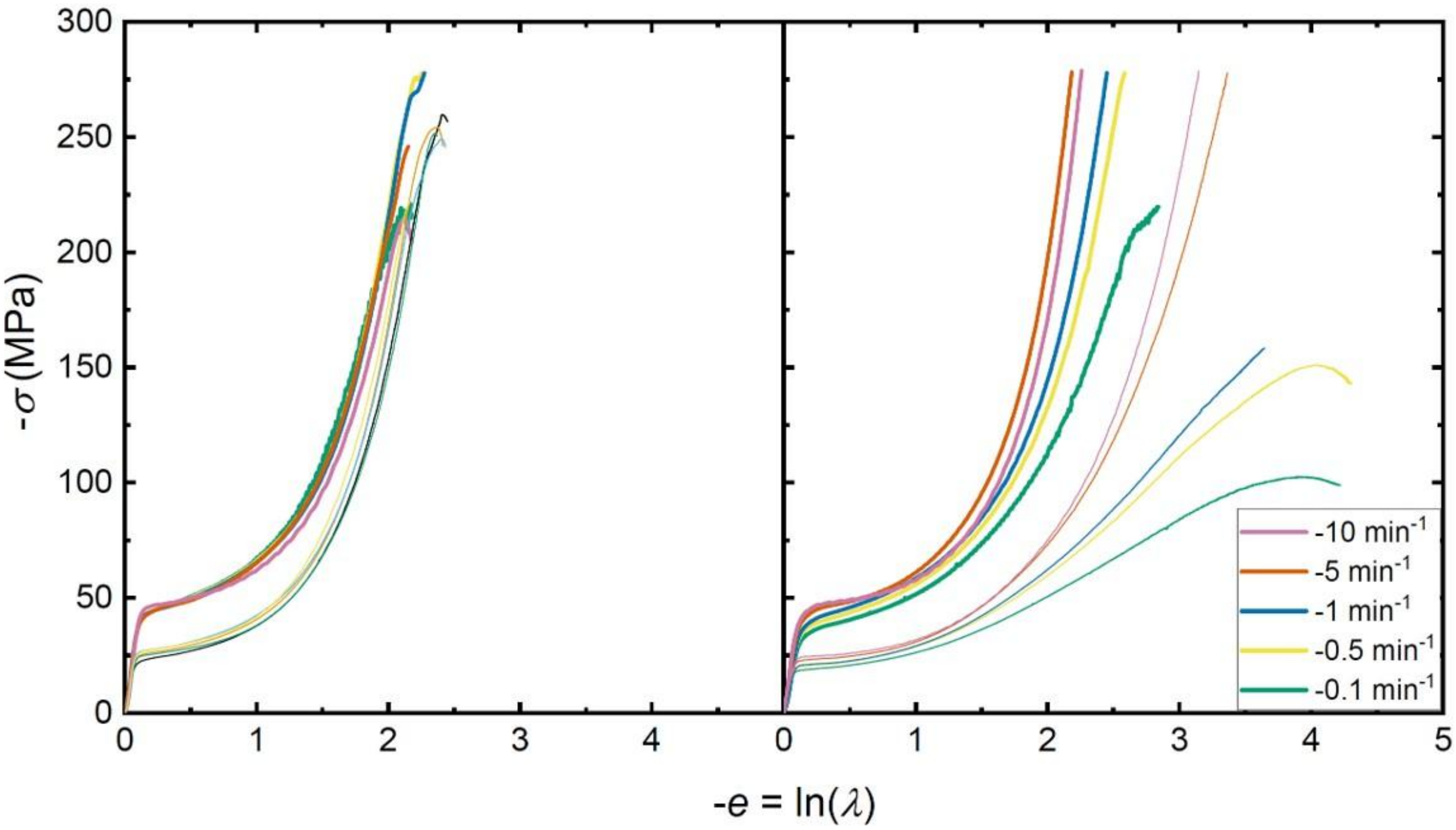


Supplementary Figure 2: Exemplary continuous true stress vs. true strain curves of a) PCL and b) PEO100 at -10 °C. Measurements were taken at the indicated constant true strain rates. For comparison the data taken at 30 °C are shown with thinner lines.

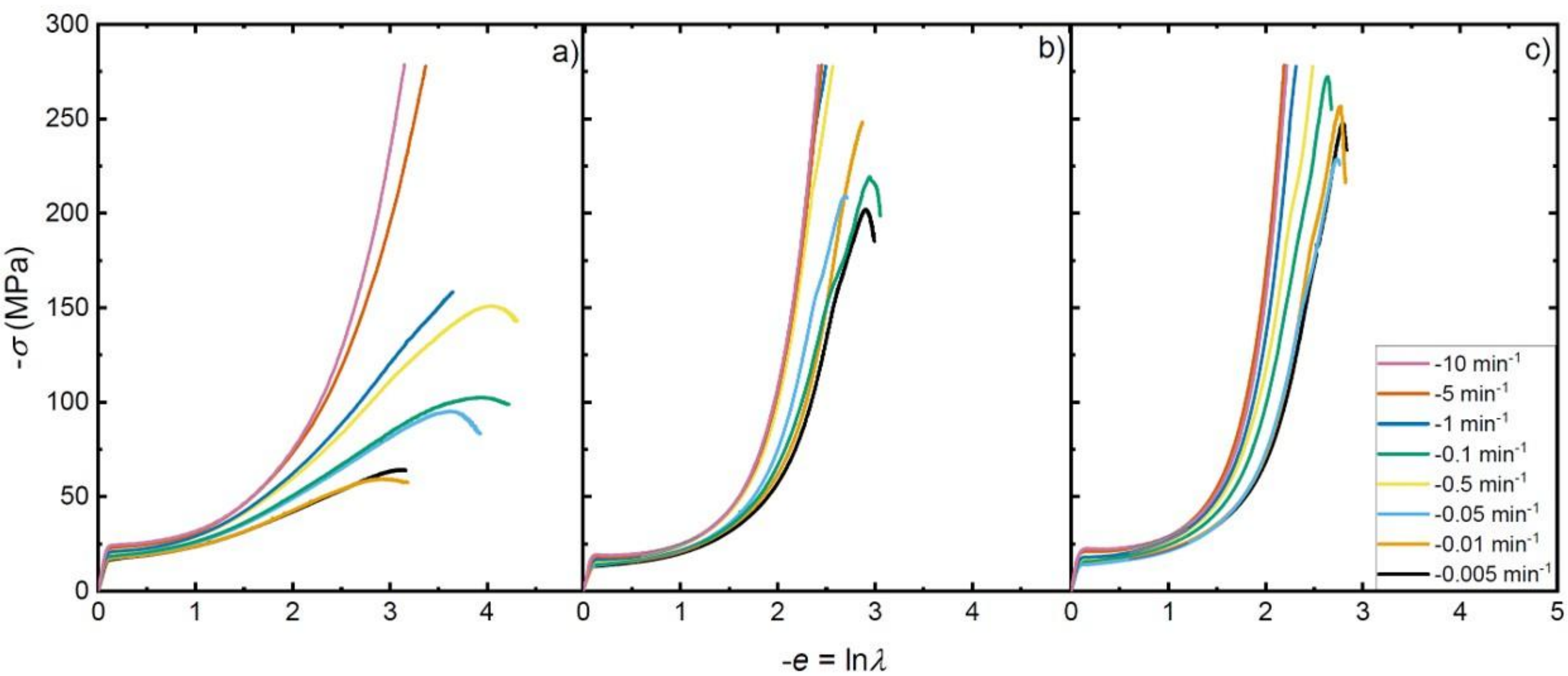


Supplementary Figure 3: Exemplary continuous true stress vs. true strain curves of a) PEO100, b) PEO400, and c) PEO900 at 30 °C. Measurements were taken at the indicated constant true strain rates.
