# Supplementary material for: The effects of intracrystalline chain diffusion on the non-linear mechanics of semicrystalline polymers
Source: Nat Commun. 2026 Sep 26;17:10235. doi: 10.1038/s41467-026-78014-w (PMC13615967; doi:10.1038/s41467-026-78014-w)
Supplement: Supplementary file 2 [file 41467_2026_78014_MOESM2_ESM.pdf]

nature portfolio

Peer Review File

The effects of intracrystalline chain diffusion on the non-linear mechanics of semicrystallinen polymers

Corresponding Author: Professor Thomas Thurn-Albrecht

Parts of this Peer Review File have been redacted as indicated to maintain the confidentiality of unpublished data.

This file contains all reviewer reports in order by version, followed by all author rebuttals in order by version.

Version 0:

Reviewer comments:

Reviewer #1

(Remarks to the Author)
In this work, the authors systematically investigated the influence of intracrystalline chain diffusion (ICD) on the non-linear mechanical stress-strain curves of two model semicrystalline polymers – poly(ethylene oxide) (PEO, fast ICD) and poly(ε-caprolactone) (PCL, extremely slow ICD) , by using plane-strain compression in combination with a stress-decomposition approach. The authors find that the network stress of PCL essentially follows neo-Hookean behavior and shows no significant rate or temperature dependence, whereas the network stress of PEO exhibits a pronounced rate-sensitive relaxation that depends on the temperature and molecular weights, suggesting the importance of the whole-chain diffusivity. The paper attributed these observed differences to different ICD activity and provided a new perspective for understanding the microscopic mechanisms of large deformation behaviors of semicrystalline polymers. The work addressed the important issue in the mechanical properties of semi-crystalline polymer materials. In comparison to the references, the work is original and holds the significance in the field of materials sciences. The experimental design and the data quality are generally good and meet the expectation of the field. The findings reveal a key role of ICD in the non-linear mechanics of semicrystalline polymers and have clear potential for publication in Nature Communications after the following-mentioned revisions are addressed. I recommend minor revision before publication in Nature Communications. Please find below my specific suggestions.

1. Figure 1 could be better added with some cartoons to illustrate the semi-crystalline texture. This could help the readers to imagine what the paper talks about.

2. Caption of Figure 2 would be better to add some explanation on the experiment method and the symbols used in the plots. Also, what was PEO^100? Was 100 necessary to occur here, whose location might be confused with the reference number? It should be explained when it occurs in the first time.

3. The color curves should be properly labeled for the case of black/white printing.

4. It would be helpful to show some morphological observations on the fracture surfaces of two samples for the conventional mechanical analysis of ductile and brittle features of the solid materials.

Reviewer #2

(Remarks to the Author)
I co-reviewed this manuscript with one of the reviewers who provided the listed reports. This is part of the Nature Communications initiative to facilitate training in peer review and to provide appropriate recognition for Early Career Researchers who co-review manuscripts.

Reviewer #3

(Remarks to the Author)

This manuscript investigates the effect of intracrystalline chain diffusion (ICD) on the non-linear mechanical behavior of semicrystalline polymers by comparing PEO and PCL under plane-strain compression. The authors conclude that the stress can be separated into elastoplastic and network contributions, and that PEO exhibits strong rate-, temperature-, and molecular-weight-dependent network relaxation, whereas PCL does not. The topic is interesting and potentially relevant to the mechanics of semicrystalline polymers. However, the mechanistic interpretation is not yet sufficiently supported, and several key assumptions in the analysis require further justification. Therefore, the reviewer recommends major revision before publication.

Some comments:
1. In this article, the authors posit that the network relaxation of PEO originates from the full-chain motion induced by ICD, whereas this is not the case for PCL. However, the authors merely conducted a fitting analysis based on stress-strain curves, neglecting the influence of crystallinity.
2. The authors are advised to conduct a study on or use the literatures to discuss the structural changes of strain-induced PCL and PEO using WAXS and SAXS, exploring the multi-structural deformation to support the author's conclusions.
3. In Line 143 on Page 7, the authors assume that beyond yielding, the additional deformation is almost entirely accommodated by the plastic element, while the elastic element remains at a fixed extension, so that the stress contribution from this branch (σ_{ep}) becomes strain-independent. After yielding, processes such as lamellar fragmentation, crystal slip, and even partial melting/reorganization may occur, which could continuously alter the elastoplastic contribution rather than keeping it constant. Such structural evolution is highly relevant to the interpretation of σ_{ep} and should not be neglected. In this regard, WAXS measurements would be highly valuable, as they could directly reveal the crystal fragmentation and orientation evolution during deformation, thereby providing structural support for the validity of the stress decomposition adopted here.
4. In Page 17, Lines 306–317, the authors attribute the suppressed relaxation at higher molecular weight to a whole-chain motion process rather than a local rearrangement. This is an important argument, but the current discussion remains too qualitative. Molecular weight does not only affect chain relaxation dynamics, but may also influence entanglement density, crystallization behavior, and the initial semicrystalline morphology. In addition, the sample preparation conditions for different PEO molecular weights are not identical (Page 20, Lines 362–369), which may further introduce morphological differences. Therefore, the observed trend cannot be attributed uniquely to whole-chain-scale ICD dynamics without a more careful discussion of these factors.

Reviewer #4

(Remarks to the Author)
Comments to NCOMMS-26-0256516

This paper presents valuable results that provide a deeper and more fundamental understanding of the mechanical properties of semicrystalline polymers.
Authors focused on mechanical behavior of two polymers, representing a so-called crystal-fixed and crystal-mobile polymers - PCL and PEO, respectively - and found fundamental differences in their intrinsic mechanical properties, which apparently depend on time-scale of the α_c relaxation and related intracrystalline chain diffusion (ICD), nearly absent in PCL and fast in PEO in the explored experimental range. This ICD strongly affects the stress response at large strains, where the amorphous network dominates the mechanical response. Consequently, fast ICD enables large deformations of the material at considerably lower stresses at small deformation rates. Authors expect these differences to be of general validity.
The paper can be of great interest to a wide audience and, in my opinion, can be published in Nature Communications after a minor revision, according to the comments below.

Comments:
1. PEO is a water-soluble polymer, so samples may be sensitive to moisture, which can noticeably influence their mechanical response, especially that associated with the amorphous phase. Did the authors take any steps to avoid moisture-related effects?

2. P.10, line191: The difference in elastoplastic stress of PCL and PEO, roughly independent of the deformation rate, can also be a simple consequence of the lower plastic resistance of PEO crystals, determined by the critical shear stress of the easiest slip system, related to the structure of crystals, but this obviously requires further investigation.

3. Figure 6: were the values of the plateu modulus of the melt G_n^m obtained experimentally in this work or were taken from the literature? If so, please provide the reference.

4. P.12, line 219: Alternatively, this can be a result of higher network density in the solid sample than in the melt due to entanglements that were redistributed to the amorphous phase rather than being resolved during crystallization, which effect may be stronger in the less mobile PCL than in PEO (mentioned in line 266). Of course, small crystallites can still contribute.

5. P.15, line 270: It may be emphasized that Gn* is analyzed as a function of strain by writing Gn*(λ) or Gn*(e) in Eq.(7)

6. References: #17, 25, 31, 32, 33,36 – Only the first author is displayed. Please, check whether this format complies with the journal’s approved style.

Version 1:

Reviewer comments:

Reviewer #1

(Remarks to the Author)
The authors have made satisfying revisions and addressed my concerns. I have no more comment and recommend to accepting the updated paper.

Reviewer #2

(Remarks to the Author)
I co-reviewed this manuscript with one of the reviewers who provided the listed reports. This is part of the Nature Communications initiative to facilitate training in peer review and to provide appropriate recognition for Early Career Researchers who co-review manuscripts.

Reviewer #3

(Remarks to the Author)
The revised manuscript is suitable for publication.

Reviewer #4

(Remarks to the Author)
I accept the Author's response to my comments and the associated changes introduced to the manuscript. I believe that the revised version of the paper is acceptable for publication in Nature Communications.

Open Access This Peer Review File is licensed under a Creative Commons Attribution 4.0 International License, which permits use, sharing, adaptation, distribution and reproduction in any medium or format, as long as you give appropriate credit to the original author(s) and the source, provide a link to the Creative Commons license, and indicate if changes were made.
In cases where reviewers are anonymous, credit should be given to 'Anonymous Referee' and the source.
The images or other third party material in this Peer Review File are included in the article’s Creative Commons license, unless indicated otherwise in a credit line to the material. If material is not included in the article’s Creative Commons license and your intended use is not permitted by statutory regulation or exceeds the permitted use, you will need to obtain permission directly from the copyright holder.
To view a copy of this license, visit https://creativecommons.org/licenses/by/4.0/

Response to reviewers

We thank all the reviewers for their careful review and their constructive comments that we address point by point below.

REVIEWER COMMENTS

Reviewer #1 (Remarks to the Author):

In this work, the authors systematically investigated the influence of intracrystalline chain diffusion (ICD) on the non-linear mechanical stress-strain curves of two model semicrystalline polymers – poly(ethylene oxide) (PEO, fast ICD) and poly(ε-caprolactone) (PCL, extremely slow ICD) , by using plane-strain compression in combination with a stress-decomposition approach. The authors find that the network stress of PCL essentially follows neo-Hookean behavior and shows no significant rate or temperature dependence, whereas the network stress of PEO exhibits a pronounced rate-sensitive relaxation that depends on the temperature and molecular weights, suggesting the importance of the whole-chain diffusivity. The paper attributed these observed differences to different ICD activity and provided a new perspective for understanding the microscopic mechanisms of large deformation behaviors of semicrystalline polymers. The work addressed the important issue in the mechanical properties of semicrystalline polymer materials. In comparison to the references, the work is original and holds the significance in the field of materials sciences. The experimental design and the data quality are generally good and meet the expectation of the field. The findings reveal a key role of ICD in the non-linear mechanics of semicrystalline polymers and have clear potential for publication in Nature Communications after the following-mentioned revisions are addressed. I recommend minor revision before publication in Nature Communications. Please find below my specific suggestions.

1. Figure 1 could be better added with some cartoons to illustrate the semi-crystalline texture. This could help the readers to imagine what the paper talks about.

We followed the suggestions of the reviewer and added an illustration of the mechanically relevant parts of the semicrystalline morphology plus a corresponding description in the main text.

2. Caption of Figure 2 would be better to add some explanation on the experiment method and the symbols used in the plots. Also, what was PEO^100? Was 100 necessary to occur here, whose location might be confused with the reference number? It should be explained when it occurs in the first time.

We followed the suggestions of the reviewer, i.e. changed PEO^{100} to PEO100, and explained that 100 stands for the molecular weight. The other PEO samples are treated analogously. The mode of deformation is now mentioned in the caption and the variables are explained.

3. The color curves should be properly labeled for the case of black/white printing.

Following the policy of the journal we changed the colors to a scheme adapted to the needs of colorblind readers. To clarify the effects of increasing rates on the stress-strain curves in Fig. 2b) we added an arrow indicating the direction of changes caused by increasing rates.

4. It would be helpful to show some morphological observations on the fracture surfaces of two samples for the conventional mechanical analysis of ductile and brittle features of the solid materials.

Although there are interesting effects, we would not like to show any pictures from fractured samples and fractures surfaces as this topic is beyond the scope of this manuscript and requires further study. While the behavior of both samples, PCL and PEO should be classified as ductile, there are systematic differences in the fracture pattern occurring at large strain as shown below. To explain our decision, we show below some preliminary results.

Redacted

Reviewer #2 (Remarks to the Author):

I co-reviewed this manuscript with one of the reviewers who provided the listed reports. This is part of the Nature Communications initiative to facilitate training in peer review and to provide appropriate recognition for Early Career Researchers who co-review manuscripts.

Reviewer #3 (Remarks to the Author):

This manuscript investigates the effect of intracrystalline chain diffusion (ICD) on the nonlinear mechanical behavior of semicrystalline polymers by comparing PEO and PCL under plane-strain compression. The authors conclude that the stress can be separated into elastoplastic and network contributions, and that PEO exhibits strong rate-, temperature-, and molecular-weight-dependent network relaxation, whereas PCL does not. The topic is interesting and potentially relevant to the mechanics of semicrystalline polymers. However, the mechanistic interpretation is not yet sufficiently supported, and several key assumptions in the analysis require further justification. Therefore, the reviewer recommends major revision before publication.

Some comments:
1. In this article, the authors posit that the network relaxation of PEO originates from the full-chain motion induced by ICD, whereas this is not the case for PCL. However, the authors merely conducted a fitting analysis based on stress-strain curves, neglecting the influence of crystallinity.
We put less emphasis on the crystallinity of the investigated samples as the influence of this quantity on the network stress, which is in the focus of our work, is small. However, we understand the concern of the reviewer and included additional information at several points in the revised version of the manuscript, in order to make the limited role of crystallinity clearer. Additionally, we added a table giving the crystallinities of all samples in the method section. Generally, the crystallinity of PCL is lower than the crystallinity of PEO. This is a typical difference between crystal-fixed and crystal-mobile polymers as mentioned in the introduction. We studied these differences previously in much detail, cf. references 25 and 26.
Generally, the modulus and the elastoplastic stress beyond yield are directly related to the crystallinity. We now mention this relation explicitly in the paragraph titled ‘Analysis of stress-strain curves’ on page 9. However, a direct comparison across different polymers is difficult, since there might be intrinsic differences between different materials, as pointed out in our manuscript on page 12. On the other hand, the crystal fraction has only an indirect effect on the network stress, insofar as crystallites act as additional junction points of the network as

discussed on page 14 of our manuscript already in the original version. However, the relation between initial network modulus and crystallinity is complex since the crystal junction points have a dynamic character for a crystal-mobile polymer as visible in the temperature and molecular weight dependence for PEO. We added a corresponding remark on page 19.

2. The authors are advised to conduct a study on or use the literatures to discuss the structural changes of strain-induced PCL and PEO using WAXS and SAXS, exploring the multi-structural deformation to support the author's conclusions.
We think an analysis of the structural changes by WAXS and SAXS cannot give further support for the conclusions of our manuscript. Our paper deals with an analysis of stress-strain measurements and focuses on the effect of intracrystalline chain diffusion on the network stress. While scattering experiments can give information about structural changes of the crystal fraction of the samples and the semicrystalline morphology, they will not give any information about the individual stress components accompanying the deformation and also no information about chain stretching, which is the origin of the network stress component. On the other hand, it is well known that during tensile deformation as well as in plane strain compression, the microstructure of semicrystalline polymers is transformed from an initial isotropic spherulitic structure to a final oriented fibrillar structure. This fact is mentioned in our introduction. We do not deny that a specific comparison of these structural changes for the two polymers investigated here might be of interest, but this is not the topic of our manuscript and rather a separate question.

3. In Line 143 on Page 7, the authors assume that beyond yielding, the additional deformation is almost entirely accommodated by the plastic element, while the elastic element remains at a fixed extension, so that the stress contribution from this branch (σep) becomes strain-independent. After yielding, processes such as lamellar fragmentation, crystal slip, and even partial melting/reorganization may occur, which could continuously alter the elastoplastic contribution rather than keeping it constant. Such structural evolution is highly relevant to the interpretation of σep and should not be neglected. In this regard, WAXS measurements would be highly valuable, as they could directly reveal the crystal fragmentation and orientation evolution during deformation, thereby providing structural support for the validity of the stress decomposition adopted here.
This question and our answer is related to the previous question. We think the comments of the reviewer are based on a misunderstanding of the approach and experimental design of our work. Certainly, beyond yielding complex microscopic structural processes do take place during deformation in semicrystalline processes and in principle it could also well be that the elastoplastic stress does not remain constant with increasing strain. However, from structural observations, it is not possible to deduce such a dependency; these could not give additional support for our Ansatz for the stress decomposition. We therefore take a different approach in our paper. We start from well-established models in the literature and first show that our approach, which includes a strain independent elastoplastic stress component, leads to a reasonable and consistent result for the simplest case, namely PCL. Then we compare this result with the data obtained for PEO, and we identify clear differences between PCL and PEO in the range, where the network stress dominates. In a next step we perform experiments (temperature and molecular weight dependence) that can be used as a crosscheck that our conclusions are reasonable and consistent.

4. In Page 17, Lines 306–317, the authors attribute the suppressed relaxation at higher molecular weight to a whole-chain motion process rather than a local rearrangement. This is an important argument, but the current discussion remains too qualitative. Molecular weight does not only affect chain relaxation dynamics, but may also influence entanglement density, crystallization behavior, and the initial semicrystalline morphology. In addition, the sample preparation conditions for different PEO molecular weights are not identical (Page 20, Lines 362–369), which may further introduce morphological differences. Therefore, the observed trend

cannot be attributed uniquely to whole-chain-scale ICD dynamics without a more careful discussion of these factors.
We followed the suggestion by the reviewer and added a corresponding remark on page 19 of the revised manuscript. Furthermore, we gave more explanation about sample preparation in the method section. Molecular weight affects the crystallinity (thicker amorphous regions), which is now given in the table in the methods section, and it might affect entanglement density. Indeed, we studied these effects previously (ref. 25,26,28). The general features of the semicrystalline morphology (stacks of crystal lamellae, spherulitic morphology) on the other hand remain typically unchanged with increasing molecular weight. The reason for the different annealing temperature in the melt for the high molecular weight samples lies in their higher viscosity. Otherwise, the temperature program for crystallization is the same for all samples. We argue that based on the observed changes in the shape of the Mooney ratio G_n^*, the general conclusion concerning the nonlocal character of the relaxation is justified, even after taking into account the additional effects mentioned by the reviewer.

Reviewer #4 (Remarks to the Author):

Comments to NCOMMS-26-0256516

This paper presents valuable results that provide a deeper and more fundamental understanding of the mechanical properties of semicrystalline polymers.
Authors focused on mechanical behavior of two polymers, representing a so-called crystal-fixed and crystal-mobile polymers - PCL and PEO, respectively - and found fundamental differences in their intrinsic mechanical properties, which apparently depend on time-scale of the α_c relaxation and related intracrystalline chain diffusion (ICD), nearly absent in PCL and fast in PEO in the explored experimental range. This ICD strongly affects the stress response at large strains, where the amorphous network dominates the mechanical response. Consequently, fast ICD enables large deformations of the material at considerably lower stresses at small deformation rates. Authors expect these differences to be of general validity.
The paper can be of great interest to a wide audience and, in my opinion, can be published in Nature Communications after a minor revision, according to the comments below.

Comments:
1. PEO is a water-soluble polymer, so samples may be sensitive to moisture, which can noticeably influence their mechanical response, especially that associated with the amorphous phase. Did the authors take any steps to avoid moisture-related effects?
All our samples were prepared under vacuum in a so-called MeltPrep device as described in the experimental section. Moisture effects should be excluded at this point. We further examined differences in mechanical properties immediately after preparation and a week later, after storage in open-atmosphere containers. We did not find any significant difference in the overall behavior of the samples. More specifically, the rate-dependent stress-strain curves followed the same trend regardless of the time between molding and testing. All further experiments were performed within one week after preparation.

2. P.10, line191: The difference in elastoplastic stress of PCL and PEO, roughly independent of the deformation rate, can also be a simple consequence of the lower plastic resistance of PEO crystals, determined by the critical shear stress of the easiest slip system, related to the structure of crystals, but this obviously requires further investigation.
We agree on this point and formulated the corresponding paragraph in our manuscript more carefully.

3. Figure 6: were the values of the plateau modulus of the melt G_n^m obtained experimentally in this work or were taken from the literature? If so, please provide the reference.
We provided the reference in the revised version of the manuscript.

4. P.12, line 219: Alternatively, this can be a result of higher network density in the solid sample than in the melt due to entanglements that were redistributed to the amorphous phase rather than being resolved during crystallization, which effect may be stronger in the less mobile PCL than in PEO (mentioned in line 266). Of course, small crystallites can still contribute.
We disagree on this point. As worked out in the given reference by Bartczak (re. 27), the network modulus is proportional to the number of network strands per total sample volume, i.e. a redistribution of the entanglements to the amorphous regions alone should not change the value of G_n. It is also of interest in this context that we know from previous experiments that entanglements are not dissolved during crystallization of PCL of higher molecular weight. We added the corresponding reference. In conclusion, we think that indeed our observation is a strong indication that crystals act as additional junction points. We would therefore like to keep the corresponding text as is.

5. P.15, line 270: It may be emphasized that Gn* is analyzed as a function of strain by writing Gn*(λ) or Gn*(e) in Eq.(7)
We followed the reviewers suggestion and modified eq. 6 in analogous way ($\sigma_n(e)$).

6. References: #17, 25, 31, 32, 33,36 – Only the first author is displayed. Please, check whether this format complies with the journal's approved style.
It does comply with the style of the journal. We used the 'Nature Communications' style for the references.
